# The Utility of Mitochondrial and Y Chromosome Phylogenetic Data to Improve Correction for Population Stratification

**DOI:** 10.3389/fgene.2012.00301

**Published:** 2012-12-21

**Authors:** Robert Makowsky, Qi Yan, Howard W. Wiener, Michael Sandel, Brahim Aissani, Hemant K. Tiwari, Sadeep Shrestha

**Affiliations:** ^1^Department of Biostatistics, School of Public Health, University of Alabama at BirminghamBirmingham, AL, USA; ^2^Department of Epidemiology, School of Public Health, University of Alabama at BirminghamBirmingham, AL, USA

**Keywords:** phylogeny, PCA, Y chromosome, mitochondria, population sub-structure

## Abstract

Genome-wide association (GWA) studies have become a standard approach for discovering and validating genomic polymorphisms putatively associated with phenotypes of interest. Accounting for population structure in GWA studies is critical to attain unbiased parameter measurements and control Type I error. One common approach to accounting for population structure is to include several principal components derived from the entire autosomal dataset, which reflects population structure signal. However, knowing which components to include is subjective and generally not conclusive. We examined how phylogenetic signal from mitochondrial DNA (mtDNA) and chromosome Y (chr:Y) markers is concordant with principal component data based on autosomal markers to determine whether mtDNA and chr:Y phylogenetic data can help guide principal component selection. Using HAPMAP and other original data from individuals of multiple ancestries, we examined the relationships of mtDNA and chr:Y phylogenetic signal with the autosomal PCA using best subset logistic regression. We show that while the two approaches agree at times, this is independent of the component order and not completely represented in the Eigen values. Additionally, we use simulations to demonstrate that our approach leads to a slightly reduced Type I error rate compared to the standard approach. This approach provides preliminary evidence to support the theoretical concept that mtDNA and chr:Y data can be informative in locating the PCs that are most associated with evolutionary history of populations that are being studied, although the utility of such information will depend on the specific situation.

## Introduction

Genome-wide association (GWA) studies have become common practice in the effort to elucidate relationships of genetic markers to many human diseases and phenotypes. While such studies may provide useful results, their usefulness depends on a careful accounting of potential confounding effects of population sub-structure. Unrecognized population sub-structure or stratification can cause misleading results in the form of spurious associations between SNPs and outcome (Type I error; Marchini et al., 2004) as well as reduced power to detect stratification specific signals (Tian et al., 2008). Fortunately, statistical methods have been developed to correct for these issues (Price et al., 2010).

Some of the various measures researchers have proposed to adjust for population stratification include genomic control, structured association, principal component analysis (PCA), multidimensional scaling (MDS), and stratification score (Bouaziz et al., 2011). The most common approach has been to use PCA on thousands of autosomal SNPs to assess genome-wide population structure. In this approach, the components are evaluated based on a scree plot (Pritchard et al., 2000; Price et al., 2006) and those deemed important are included as covariates in the association models. There are several PCA-based approaches, but EIGENSTRAT is widely used in GWA studies (Price et al., 2006). The utility of these approaches depends on many assumptions, mainly the degree of stratification where population structures can be discrete, admixed, or a combined hierarchal (Bouaziz et al., 2011). Recently, there has been a discussion of whether population stratification information should be included as random or fixed effects in subsequent analyses (Price et al., 2010), but either approach relies on the components actually accounting for the population sub-structure. Some statistical approaches, such the Bayesian LASSO (De Los Campos et al., 2009), which include all the markers simultaneously, implicitly incorporate information pertaining to population structure in a manner similar the PCA approach, although in a regularized manner. While ideal from a predictive standpoint, the Bayesian LASSO is less insightful for standard analyses aimed at quantifying the underlying biology. Recently, researchers have searched for better ways to determine which components are “important” (Li et al., 2010). The approach of Li et al. combined the results from MDS and a phylogenetic analysis and found they were better able to capture population stratification. Overall, the literature is quite rich in extensions of methods to account for each type of population stratification (Tian et al., 2008; Tiwari et al., 2008; Zhang et al., 2008); however, there is no gold standard that can be applied to all stratification scenarios.

To understand the relationship between population sub-structure and genetic makeup, autosomal SNPs are routinely used to estimate individual ancestry, whereas markers in the mitochondrial DNA (mtDNA) and Y chromosome (chr:Y) are used to scale inferences of human evolutionary history. mtDNA and chr:Y markers have also been applied independently to forensic, sex-biased admixture studies as well as other migration and population genetic studies (Bryc et al., 2010; Lao et al., 2010; Simonson et al., 2011; Stefflova et al., 2011); however, it is unknown whether these markers could add any additional ancestral information to the current autosomal PCA approach used to account for population stratification. Typically, scree plots based on Eigen values of PCAs are used to determine the number of components to include for adjustments, although this approach is subjective across studies. Phylogenies constructed using mtDNA and chr:Y data could help elucidate evolutionary population structure of humans; specifically, the recovered clades that have statistical support could be categorized and compared to PCA component grouping to determine more objectively which components should be included to account for realized population structure. Unlike autosomal and X chromosomes, which can recombine and thus create diversity in the haplotypes, mtDNA, and chr:Y are based on single DNA strands and represent the genetic makeup of a distant ancestor. For example, if a single male migrated between two separate populations, the evidence for this could be easily recognized in his male descendents over generations by examining the chr:Y markers, while a signal based on autosomal data would be ambiguous. The smaller effective population size of mtDNA and chr:Y markers versus autosomal markers due to their haploid nature and single-sex inheritance also increases the likelihood of an informative phylogeny due to the reduced coalescent time of any two randomly drawn sites (Moore, 1995). Therefore, our method differs from Li et al. (2010) by using mtDNA and chr:Y data to construct the evolutionary tree, using a phylogenetic method as opposed to similarity method (neighbor-joining), and considering only significantly supported nodes.

In the present study, we examined whether a phylogeny based on mtDNA and chr:Y markers could contribute toward correction of population stratification. This also allows us to examine whether mtDNA and chr:Y data could further distinguish sub-populations within relatively homogenous populations such as Caucasians (i.e., we assume that association studies can be confounded by further unknown sub-structure). On the other hand, the information can also be important in identifying disease-associated variants that could differ within the sub-structures such as admixed sub-populations. For example, the African-American population is quite heterogeneous based on autosomal PCA but any additional information from mtDNA and chr:Y markers could help in accurately accounting for their dynamic population histories. Here, we determined the relationship of genetic ethnicity as derived from the PCA of autosomal SNPs to a mtDNA and chr:Y based phylogenetic tree.

## Materials and Methods

We analyzed autosomal, mtDNA, and chr:Y marker data from 234 males, 45 Caucasians, 23 Chinese, 23 Japanese, and 53 Yorubans from the parent HapMap population (data released August, 2010, on NCBI B36 assembly, dbSNP b126 – mtDNA and chr:Y data were only available from the subset and not entire HapMap participants) and another 90 healthy African-Americans from another ongoing population genetics analysis program (PopGen) project (NCT00119067), based on an anthrax vaccine trial, as previously described (Pajewski et al., 2012). Briefly, NCT00119067 randomly enrolled healthy individuals to receive Anthrax Vaccine Adsorbed AVA and the goal of the parent study was to determine the influence of genetic variants on differential antibody response to AVA. Since the participants were healthy with no known disease outcomes, it serves as a representative population. African-Americans were defined by a principle component analysis (PCA) based on the SNPs from Affymetrix Genome-Wide 6.0 array (Affy 6.0 array). To have consistent SNPs across all populations, we limited the analysis to the same 901,678 SNPs (Affy 6.0 array) in the HapMap population^1^. Of note, since HapMap used multiple genotyping platforms, genotype calls had to be accurately matched for the DNA strand. We excluded the autosomal SNPs that required such correction, but to maximize the mtDNA and chr:Y SNPs, as a quality control measure, we matched the allele frequencies among all Caucasians (*n* = 174) in the overall HapMap and the PopGen cohort (*n* = 794) to determine the major and minor allele in two mtDNA and 48 chr:Y markers. Most minor alleles were rare, and overall frequencies were <30% for all minor alleles in these SNPs, providing confidence in our assignment. After filtering the inconsistencies^2^ in the HapMap and PopGen GWA data (Pajewski et al., 2012), there were 505,734 autosomal, 106 mtDNA, and 252 chr:Y overlapping SNPs for all five populations.

**Figure 1:**
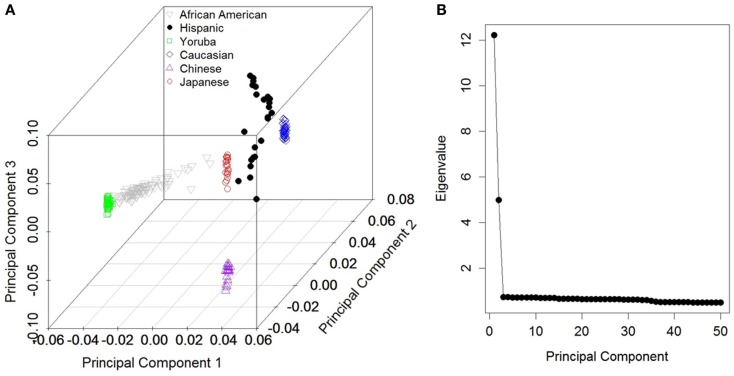
**(A)** First three principal component scores based on all autosomal markers with *post hoc* groupings. **(B)** Scree plot depicting Eigen values for the first 50 components for the corresponding components in **(A)**.

In order to uncover the population structure and define race-based sub-populations, we first performed a PCA implemented in the Eigenstrat software (Price et al., 2006). The principal components were derived from the genotype data on all 505,734 autosomal SNPs (mtDNA and chr:Y markers were excluded). Linear discriminant analysis (Rao, 1973), using the Discrim procedure in SAS version 9.2 (Cary, NC, USA), was performed to decompose the populations. The Eigen values from the first 50 principal components were visualized in a scree plot (Figure 1).

SNP data for the chr:Y, mtDNA, and a concatenated dataset (chr:Y and mtDNA combined) were analyzed using the parsimony optimality criterion in TNT 1.1 (Goloboff et al., 2008). When mtDNA and chr:Y were combined, there were no redundant haplotypes, suggesting unique combinations for each individual. Parsimony analyses were run using a New Technology search implementing the ratchet, drift, and tree fusing options with default settings (Goloboff, 1999; Nixon, 1999). Briefly, the New Technology search consists of improved heuristic search algorithms that dramatically decrease computational time and allow for large data matrices. One-thousand random addition sequences were performed, and seven equally most-parsimonious trees (each requiring 1,088 steps) resulting from the combined mtDNA and chr:Y analysis were merged into a strict consensus tree. Sample CHE-15 was the designated out-group. To assess nodal support, 1,000 bootstrap replications were performed using the same setting as above. All nodes lacking statistical support based on bootstrap proportion (<70) percent or containing less than five individuals were collapsed, and each remaining node was give a unique identifier.

To determine whether phylogenetic data can be useful toward selecting components, we performed separate subset logistic regressions for each clade (a clade is the set of descendants from a specific node), where phylogenetic position (within clade/outside clade) was coded as binary. Using clade inclusion as the response variable, we performed best subset regression using the “bestglm” package in R 2.14.0^3^. We opted to evaluate models using fivefold cross validation, with 1,000 replicates, where the best model is the one with the fewest parameters that is within 1 SD of the model with the absolute best predictive accuracy (Hastie et al., 2009), based on means squared error in the test sample. For each clade, the “important” principal components were used as predictors in a multiple logistic regression and each component’s β, SE, and *p*-value were estimated.

To determine the extent to which our approach is able to reduce Type I error, we simulated null phenotypic data that was correlated with population structure. We randomly evolved a continuous character 15 times for each of the seven best trees inferred from Y and mt data combined (a total of 105 simulated characters), using the default Brownian motion model in Mesquite 2.75 (Maddison and Maddison, 2011). Briefly, the Brownian motion model is described as a stochastic model in which expected change is distributed normally with mean zero and variance proportional to branch length times a constant rate parameter (assuming a rate of one). We then ran standard regressions for each SNP across all 105 simulated phenotypes using both the standard approach guided by a scree plot and our phylogenetically guided approach. To determine the components that are most associated with evolutionary history, we examined Table 1 and counted the number of times that a component was chosen as an important predictor across clades. While any cutoff could be included, we used count cutoffs of five (Our Method 1) and three (Our Method 2) to determine variable inclusion. Analyses included models with (a) no covariates (Null model), (b) the first two principal components (Standard correction), (c) the 1st, 2nd, 33rd, and 34th components (Our Method 1), and (d) 1st, 2nd, 4th, 5th, 33rd, 34th, and 35th components (Our Method 2). For each SNP, we calculated the *p*-value associated with each analysis and used them to determine the relative Type I error inflation. Type I error rates were compared three ways; first, by calculating the η statistic in the “fdrtool” package, which measures how well the *p*-values fit a uniform distribution; second, by calculating the number of SNPs that would have associated *q*-values (Storey, 2002) <0.20; third, by calculating number of SNPs that would have associated local FDR values <0.20 (Benjamini and Hochberg, 1995). For each simulated dataset, we counted the number of false positives based on the *q* and FDR approach. We then combined the 105 false positive counts based on FDR and *q*-values and calculated the median number of false positive counts for each adjustment approach. Given the non-independence of the 105 simulated datasets, we were unable to perform statistical tests to compare the methods.

**Table 1:** **Principal Components based on autosomal markers and associated phylogenic clades of mitochondrial DNA (mtDNA) and Y chromosome chr:Y markers**.

Principal components	Mitochondrial and Y chromosome (chr:Y) markers based phylogenic clades									
	X1	X11	X113	X2	X21	X211	X2111	X2112	X21121	X21122
1st	E^−157^	E^−41^	.	E^−157^	E^−85^	E^−71^	E^−7^	E^−43^	E^−31^	E^−22^
2nd	E^−17^	.	.	E^−17^	E^−18^	E^−20^	E^−14^	.	E^−26^	E^−53^
3rd	.	.	.	.	.	.	.	.	.	E^−4^
4th	.	.	.	.	.	.	E^−11^	E^−6^	.	E^−15^
5th	.	.	.	.	.	.	E^−8^	E^−3^	.	E^−8^
9th	.	E^−3^	.	.	.	.	.	.	.	.
11th	.	.	.	.	.	.	E^−5^	.	.	E^−6^
12th	.	.	.	.	.	.	.	.	.	E^−3^
14th	.	E^−3^	.	.	.	.	.	.	.	.
18th	.	E^−3^	.	.	.	.	.	.	.	.
25th	.	E^−5^	.	.	.	.	.	.	.	.
27th	.	E^−3^	.	.	.	.	.	.	.	.
29th	.	E^−3^	.	.	.	.	.	.	.	.
33rd	E^−15^	E^−42^	E^−4^	E^−15^	.	.	.	.	.	E^−3^
34th	E^−6^	E^−21^	.	E^−6^	E^−19^	E^−15^	.	E^−9^	.	.
35th	E^−6^	E^−30^	.	E^−6^	.	.	.	.	.	.

*Components selected based on subset selection reported as the corresponding *p*-values (only the decimal place position is reported) based on *t*-statistics. Components that were not significantly associated with any group (*n* = 34) as well as groups that had no predictive components (*n* = 9) are not displayed in the table*.

## Results

**Figure 2:**
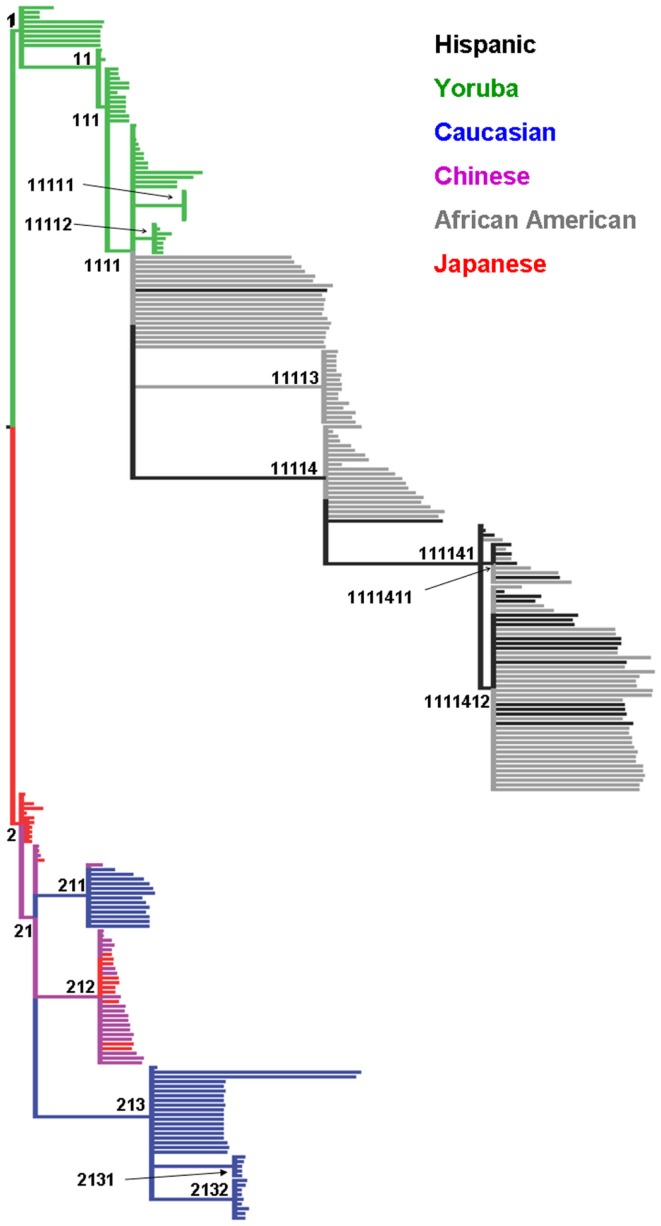
**Maximum parsimony cladogram of mitochondrial DNA (mtDNA) and Y chromosome (chr:Y) single nucleotide polymorphisms**. All nodes have >70% bootstrap proportion. Unique identifiers (*n* = 19) were assigned to each statistically significant node. Terminal branch tips are colored based on groupings assigned in Figure 1 using the same color coding (Green, Yoruba; Black, African-American; Red, Japanese; Purple, Chinese; Blue, Caucasian).

Figure 1A depicts the first three components based on the PCA using the autosomal markers and our grouping of each individual (approximately concordant with Caucasians, Africans, Asians, and African-Americans). As reported in numerous previous studies, the major ethnic groupings were well separated from one another. From the scree plot (Figure 1B), it is evident that the first two components accounted for the vast majority of variance attributable to population structure. The phylogenies produced using the mtDNA and chr:Y datasets separately were very similar (data not shown), except that less structure was present in individual analyses, so only the phylogeny from the concatenated dataset is shown (Figure 2). Because we had no “out-group” to root the tree, we rooted the tree manually in a manner that agrees with current human evolutionary understanding (Li and Durbin, 2011; Pritchard, 2011). Overall, the phylogenetic structure is as expected; African-Americans branched off of Yorubans whereas Chinese, Japanese, and Caucasians shared a common ancestor. We did recover two separate Caucasian clades (2111 and 21122), one of which (21122) is more closely related to the Asian clade 21121 than to the other Caucasians. To visualize the extent of concordance of the mtDNA and chr:Y phylogeny with the PCA groupings, we color coded each terminal branch based on the groupings assigned in Figure 1A, revealing several clades composed of individuals from the same major PCA groupings.

**Figure 3:**
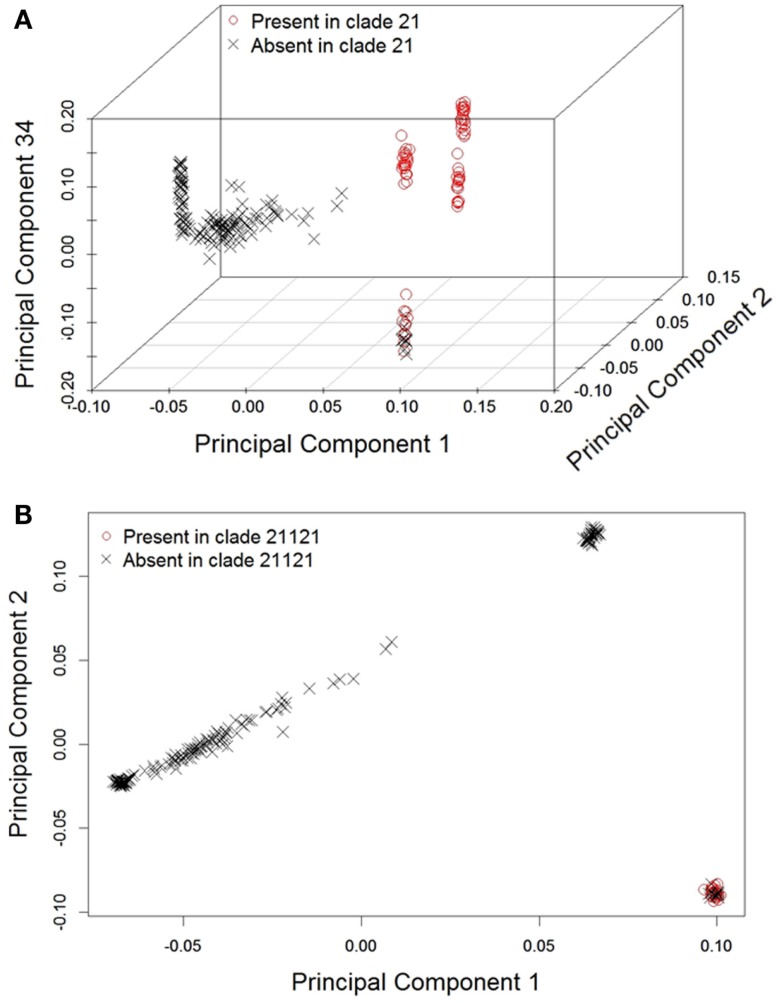
**Example plots depicting how principal component scores are associated with phylogeny: (A) 21 with principal components 1, 2, and 34 and (B) 21121 with principal components 1 and 2**.

To determine if the clades recovered in the phylogeny were concordant with any of the first 50 (1–50) principal components, we performed 19 subset logistic regressions, for which inclusion in each clade was the dependent variable. Table 1 displays the components associated with the prediction of each clade and their corresponding *p*-values. As expected, based on the scree plot, the first two components were the most important factors. This was judged based on the proportion of time each component was “important” across the clades. However, additional higher order components (e.g., 33rd–35th) were considered important for several clades. On the other hand, some components were not associated with any of the clades (e.g., 6th, 7th, 8th, etc.), demonstrating that lower order components are not necessarily most concordant with the mtDNA and chr:Y phylogenetic signal. Figure 3 visually illustrates the agreement between phylogenetic and principal component signal of two clades; 21 (Figure 3A) and 21121 (Figure 3B). Principal components 1, 2, and 34 are associated with clade 21 whereas only components 1 and 2 are associated with clade 21121.

**Figure 4:**
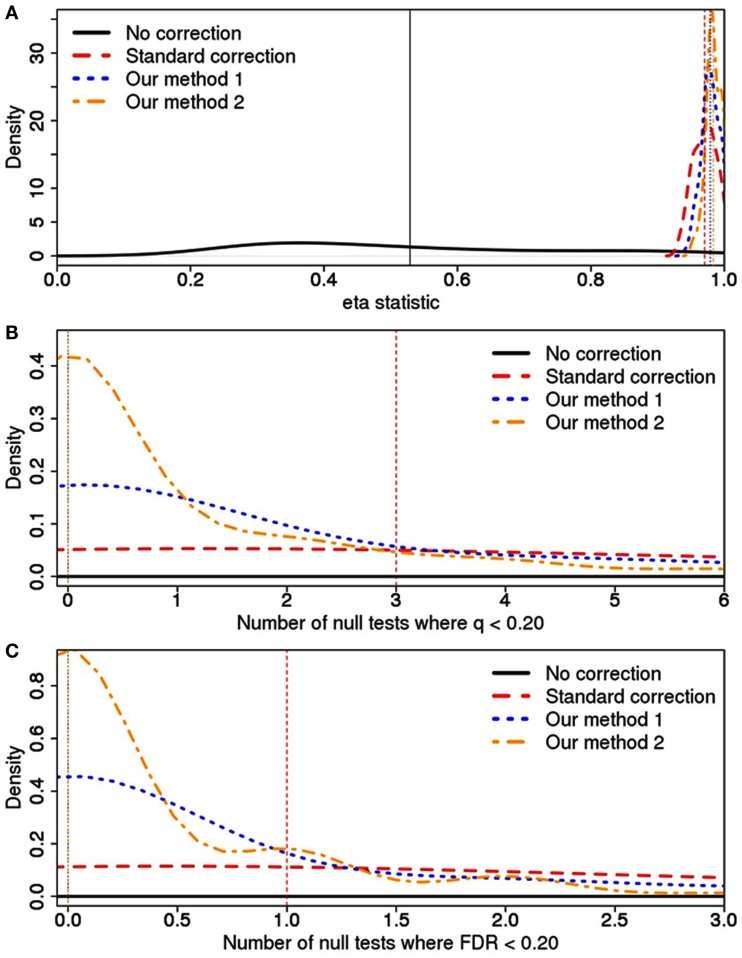
**Type I error rates and calculated using (A) the η statistic, (B) *q*-value, and (C) FDR value across various correction strategies**. For each correction type, a vertical line has been drawn at the median value, although this is off the scale for the “No correction” approach in plots **(B,C)**. Additionally, “Our methods” 1 and 2 have the same median value in plots **(B,C)**. In **(A)**, the scale on the *x*-axis is designed to demonstrate how the various correction methods all vastly outperform the model without any correction. In **(B,C)**, the *x*-axis scale has focused in on the three correction methods to better demonstrate the gain resulting from our approach.

The simulations demonstrate that our methods (1 and 2) can reduce Type I error rates, although the reduction can be minimal compared to the standard approach (Figure 4). This assessment was based on a pooling of analytic results across the 105 simulated datasets. For example, while the standard method will lead to a median of three tests with *q* < 0.20 and 1 test with FDR < 0.20, our methods both reduce this median to 0. Regardless of the manner in which we measured Type I error rates (η statistic, *q*, or FDR), analyses using the principal components defined using our approach resulted in the lowest Type I error rates.

## Discussion

We sought to determine (a) whether a phylogenetic analysis of mtDNA and chr:Y data could help further define the population structure based solely on PCA of autosomal SNPs, and (b) which principal components should be included to account for population structure. We recovered multiple significant clades using the combined mtDNA and chr:Y dataset, found that several components were strongly associated with the clades, and demonstrated using simulations that our approach leads to a better correction of population stratification (i.e., lower Type I error rates).

It is important to acknowledge that it is unnecessary to correct for population structure if the structure is not associated with the outcome of interest. It has been suggested that components should only be included if such an association is demonstrated due to the resulting loss of power (Cox and McCullagh, 1982; Yu et al., 2008). Based on the scree plot in Figure 1B, it would be advisable to include only the first two components if they are necessary with respect to the outcome. In this study, we introduced the hypothesis that significant phylogenetic structure based on mtDNA and chr:Y markers can be a guiding tool to predict additional components needed for adjustment, regardless of their associations with the outcome of interest (although this can still be considered if desired). Given that (1) the primary reason PCs are included in a GWA studies is to account for population structure and reduce Type I errors and (2) our approach is designed to locate the PCs that are most associated with evolutionary history, we feel that there exists a theoretical justification for the approach. Additionally, both our and the simulations of Price et al. (2006) demonstrate that including PCs does reduce the false positive rate. Our simulations were designed to solely determine if our method will reduce Type I error. The consensus phylogenetic tree used to define clades is highly collapsed compared to any of the most-parsimonious trees; thus it is possible that the important information may be lost and there is little residual signal. Fortunately, this does not seem to be the case, although the reduction in Type I error rates compared to the standard scree plot approach is minimal and the benefit of this approach is likely minimal in small sample sizes of homogenous populations. While we have not specifically compared the individual approaches, or any combination thereof for the maximal benefit, our results provide preliminary indications that mtDNA and chr:Y data could be informative. Future research that focuses on the various approaches would likely be very helpful, although this will be a monumental task and is beyond the scope of this paper. We do caution that the analyses were performed on Affymetrix platform based GWA data and is specific to males. Also our sample size was limited to only the parent HapMap participants since mtDNA and chr:Y data were not available for all participants. Thus future comparisons using comprehensive data in larger populations, including simulated data, may help in the evaluation of these alternative approaches; including X-chromosome data from females may also help.

One difficulty associated with this approach is determining which components should be included. Table 1 depicts how 16 components are associated with at least some of the phylogenetic structure, but the extent to which each component is concordant with phylogenetic signal varies greatly. We take the approach that the information from all clades is considered equally and that cutoff of importance of the component is three or five. Another approach would be to weight clades based on the ratio of members/non-members, such that ratios closer to 1:1 would receive the highest weight, although devising such a weighting scheme in a meaningful manner would be very difficult. Based on our equal weighted scheme, a more conservative approach is to use each component that shows any concordance with the phylogenetic signal, which would result in 16 components. Another approach is to use only the components that are represented as often as seen for the “important components,” as defined by scree plots. Based on this, since only the first two components are important based on the scree plot and a minimum of seven clades were associated with either of them, we would be limited to only those two components since the remainder of the components only had six associated clades or less. Overall, the practicality of these approaches or other hybrid approaches would depend on the relative loss of power as well as reduction in bias that results from incorporating such components in the analyses.

While previous studies have adjusted for mtDNA structure based on known haplogroups (Hudson et al., 2007; Biffi et al., 2010), we have incorporated the chr:Y data as well in order to obtain a more complete understanding of the evolutionary history. We purposefully excluded autosomal data in creating the phylogeny because its mode of inheritance obfuscates the signal perpetuated by mtDNA and chr:Y markers. Additionally, by not including autosomal markers, dependence between the datasets used to derive the principal components and phylogeny is substantially reduced. mtDNA has regularly been used in other animals to discern phylogenetic histories (Avise, 2000) and the results have often been concordant with nuclear marker analyses (Moore, 1995), both with sequence or fragment data. Previous studies have also reported that mtDNA PCs correlated well with the traditional mtDNA haplogroup assignment, but showed only limited correlation with nuclear PCs (Biffi et al., 2010). One concern with the mtDNA data from commercially available genome-wide arrays is that it does not capture all the common variants in the mitochondrial genome, specifically in the hypervariable region. However, Biffi et al. reported strong correlation between both the Illumina and Affymetrix arrays and the validated panel of 144 mtDNA markers. Thus, although this approach may not restrict generalizability and allow for merging of data from various marker sets, the data need to be interpreted cautiously when using different sets of markers.

Discordance can also occur between mtDNA/chr:Y and nuclear marker information for a number of reasons (Hoelzer, 1997; Springer et al., 2001; Shaw, 2002; Spinks and Shaffer, 2009). This leads to the question of why only moderate agreement exists between the two datasets in humans. For example, both Caucasians and Asians were subdivided into two statistically significant clades (Figure 2), but concordant divisions were not apparent in the autosomal PCA data. Sex-biased dispersal is not likely because the mtDNA (female lineage) and chr:Y (male lineage) phylogenies are very similar. Other reasons include different effective population sizes as well as enhanced dispersal ability in humans. Perhaps minimal concordance is expected between autosomal PCA and mtDNA/chr:Y phylogeny, given the differences of the two methods. If that is the case, then the concordance that is observed likely represents major population structure that should be accounted for in subsequent analyses. One may argue that simply including the clade information as a factor variable, as opposed to the PCs associated with the clades, may suffice. We feel the PCs contain more information than the clade variables, given their continuous nature. Furthermore, the orthogonal nature of the PCs, as opposed to the clades, allows the inclusion of multiple covariates without having to worry about autocorrelation.

In summary, we have presented an approach using phylogenetic data from mtDNA and chr:Y to help guide which nuclear derived principal components most account for population stratification. This method uses the maternal and paternal lineage data to determine which principal components are most associated with population stratification. Utilization of this procedure allows researchers to objectively include components that are most concordant with the evolutionary history of populations that are being studied. Further work is needed to evaluate how this method compares with other approaches, including using X-chromosome data in females, and how best to determine the appropriate components in genetic association studies.

## Conflict of Interest Statement

The authors declare that the research was conducted in the absence of any commercial or financial relationships that could be construed as a potential conflict of interest.

## Footnotes

^1^http://ftp.ncbi.nlm.nih.gov/hapmap/genotypes/2010-08_phaseII+III/00README.txt

^2^http://ftp.ncbi.nlm.nih.gov/hapmap/genotypes/2010-08_phaseII+III/inconsistencies/

^3^http://www.r-project.org/
